# Compact Elliptical UWB Antenna for Underwater Wireless Communications

**DOI:** 10.3390/mi12040411

**Published:** 2021-04-07

**Authors:** Adam R. H. Alhawari, Sama F. Majeed, Tale Saeidi, Sajid Mumtaz, Hisham Alghamdi, Ayman Taher Hindi, Abdulkarem H. M. Almawgani, Muhammad Ali Imran, Qammer H. Abbasi

**Affiliations:** 1 Electrical Engineering Department, College of Engineering, Najran University, Najran 55461, Saudi Arabia; hg@nu.edu.sa (H.A.); athindi@nu.edu.sa (A.T.H.); ahalmawgani@nu.edu.sa (A.H.M.A.); 2 Department of Electrical and Electronic Engineering, Faculty of Engineering, University Putra Malaysia, Serdang 43400, Malaysia; sama.fm4@gmail.com; 3 Electrical and Electronic Engineering Department, University Teknologi PETRONAS, Bandar Seri Iskandar 32610, Malaysia; sajid_16000529@utp.edu.my; 4 James Watt School of Engineering, University of Glasgow, Glasgow G128QQ, UK; muhammad.imran@glasgow.ac.uk (M.A.I.); Qammer.Abbasi@glasgow.ac.uk (Q.H.A.); 5 Artificial Intelligence Research Centre (AIRC), Ajman University, Ajman, United Arab Emirates

**Keywords:** UWB antennas, 5G band, underwater communications, channel capacity, bit rate error

## Abstract

The increasing needs of free licensed frequency bands like Industrial, Scientific, and Medical (ISM), Wireless Local Area Network (WLAN), and 5G for underwater communications required more bandwidth (BW) with higher data transferring rate. Microwaves produce a higher transferring rate of data, and their associated devices are smaller in comparison with sonar and ultrasonic. Thus, transceivers should have broad BW to cover more of a frequency band, especially from ultra-wideband (UWB) systems, which show potential outcomes. However, previous designs of similar work for underwater communications were very complicated, uneasy to fabricate, and large. Therefore, to overcome these shortcomings, a novel compact elliptical UWB antenna is designed to resonate from 1.3 to 7.2 GHz. It is invented from a polytetrafluoroethylene (PTFE) layer with a dielectric constant of 2.55 mm and a thickness of 0.8 mm. The proposed antenna shows higher gain and radiation efficiency and stability throughout the working band when compared to recent similarly reported designs, even at a smaller size. The characteristics of the functioning antenna are investigated through fluid mediums of fresh-water, seawater, distilled water, and Debye model water. Later, its channel capacity, bit rate error, and data rate are evaluated. The results demonstrated that the antenna offers compact, easier fabrication with better UWB characteristics for underwater 5G communications.

## 1. Introduction

The efforts to have better quality, higher speed, higher data rate transmission and low-cost systems in communication industries have been increased due to the demand of the industry [1]. Moreover, the interests are steadily escalating with rapid technological advancement. As a crucial device in communication and telecommunication technologies, antennas are required to have good performances to accelerate the communication process [2,3]. Apart from having higher speed, the communication systems are expected to overcome bulky and a large printed area problem. In addition to that, these antennas are utilized in many applications, such as telecommunication and communication technologies [4]. Distinctively that includes the underwater communications and their related devices, especially with regard to the mutual conversion of electric current to electromagnetic waves and vice versa. Moreover, this concerns their capability of transmitting and receiving the signals [5]. Fast development in the industry brought too many interests in underwater communication systems, especially when these systems were applied for localizations [6]. The localization devices and systems were utilized in many applications and for many purposes, such as exploration missions and rescue programs [5]. There are many methods used in underwater applications, like the acoustic systems, which showed many drawbacks, as listed: narrow bandwidth (BW), multi-path fading, too much delay in propagation and unwanted backpropagation [7]. Furthermore, they are broadly utilized for the applications included: seaward oil and gas field monitoring, coastline security and surveillance, and oceanographic information gathering, which will require an information return between two, which include an apparent utility list of automated underwater vehicles (AUVs) [8]. Additionally, a system was considered to enhance its output using both wired and wireless sensors [9,10]. 

However, the inevitable electromagnetic wave losses in water had influenced most of the remote networks to necessarily depend on the sound waves, which are preferred to electromagnetic waves [11]. If the sonic waves are preferred to electromagnetic waves, scientists had to use many sonic transmitter/receiver systems to cover a wider area while it could be costly in comparison with electromagnetic waves-associated systems. Furthermore, due to the costly maintenance procedure of optical and acoustic systems, some standard working in the microwave (MW) region, such as Wireless Local Area Network (WLAN) with the IEEE 802.11 standard, was used in many applications with reliability. It might be due to the ease of use and the lower cost in comparison with the other measures. ISM (Industrial, Scientific, and Medical) band is another free band that can be utilized with low cost for the WLAN applications. Furthermore, this band is used broadly in many applications, especially when the low power was required.

The current interests in underwater communications mostly focused on increasing the distance between the nodes while keeping the network in broad BW and a high data rate transition [12]. The propagation methods like acoustic, electromagnetic waves and optical communication for underwater communication showed benefits and drawbacks, which presented in Reference [8]. For instances, the ocean water contains many particles that degrade the signal level and power in an optical range when it faces the oceanic water. The matter is worse for the acoustic signals and propagations, which are more vulnerable toward the sea water particles [13,14]. 

Thus, electromagnetic signals have a better prospect specifically for short distances when higher data rate up to 10 Mbps is required. Besides, the electromagnetic waves very much depend on electrical properties of a material, such as permittivity of the environment under test [15]. The underwater communications are not only restricted to the underwater communications but also involve communications from the water surface to underwater communications, and vice versa. Regretfully, if underwater communication only is available, the sonic systems are not reliable due to being bulkier and costlier. Besides, the electromagnetic (EM) waves also showed promising outcomes [16,17,18,19,20] for other types of communication other than underwater communication, such as air-to-water, surface-to-surface, and vice versa. 

Due to the previously mentioned problems, researchers need antennas with broader BW to cover more of the frequency band while using frequency ranges beyond MW. Thus, wide bands and ultra-wideband (UWB) systems are promising alternative devices. The recent narrow-band [21,22,23,24,25] antennas with a lower gain and a bigger size are designed with a complicated structure even though the Photonic Band Gap (PBG) structure could be a promising periodic structure to compensate those drawbacks. There are wide-band and ultra-wideband antennas in [26,27,28,29,30,31,32,33] with less working BW, and a lower gain along with more substantial dimensions compared to the operating frequency for underwater communications. However, their implemented patch shape antennas complicated the design more. Therefore, opting for the elliptical shape patch simplifies the design for UWB antennas [34]. Generally, similar designs for underwater communications are very complicated to fabricate and their massive structure made them heavier. 

Researchers have tried to design an antenna to work at lower frequency bands to be more applicable for underwater communications. They designed a loop antenna, a dipole antenna, for narrowband communication [26,32], and a bow-tie antenna [35] for short-range communications, especially an ISM band [36]. However, the authors in Reference [26] designed a UWB antenna for the range of 0.15–1 GHz, which was bulky and longer-range communication as compared to the conventional UWB antennas working for short-range communications. The narrow band antenna in Reference [36] was investigated in the frequency range of 0–10 GHz. They used a two-antennas system investigating only sea water. However, the impedance BW of the antenna was not at an acceptable level. A taper slot antenna (TSA) UWB antenna was designed for underwater communication in the microwave band and investigated as a single unit and multiple unit array of the antenna. The antenna had larger dimensions as 69 × 90 mm^2^. It achieved roughly a BW of 4.3–7.8 GHz [37]. 

This paper contains four sections as follows. The first part gives an introduction and background about the related works. Then, the antenna configuration and design steps along with the simulation setup are presented in section two. The third section shows the results and discussion. Finally, the paper is concluded in part four. 

## 2. Design and Characterization of the Proposed Antenna

**Figure 1:**
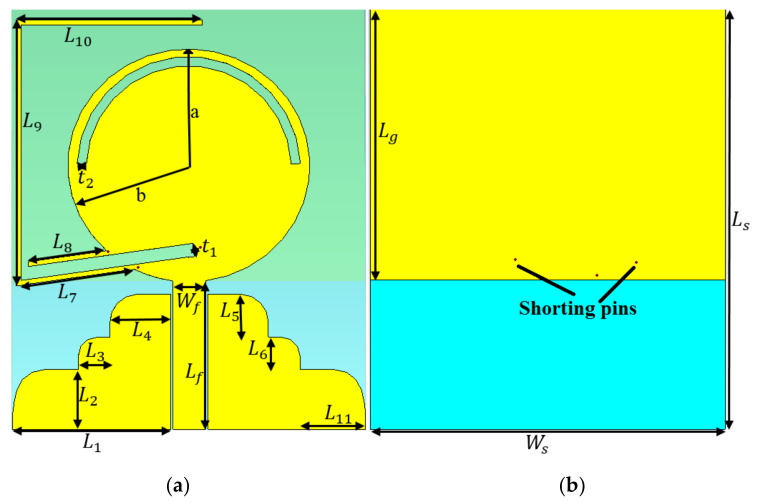
The proposed antenna’s simulated prototype: (**a**) front view and (**b**) ground view.

An ultra-wideband antenna is designed to both send and receive UWB impulses within a frequency range of 1.26 GHz to 7.2 GHz (The 3D Computer Simulation Technology (CST) microwave studio software was used to design and investigate the antenna’s characteristics in free space and water medium). It uses a patch with an elliptical shape, stub, shorting pins, and a curved staircase ground (GND), and a feed line (FL) is used to feed the antenna through a SubMiniature version A (SMA) port. Therefore, if the FL dimensions (L and W) were appropriately designed, the spurious currents are possibly ignored (Figure 1). Otherwise, these currents can cause too many distortions in the antenna’s radiation pattern and degrade their performance (The antenna is designed in a frequency range of 0–10 GHz). The UWB antennas have the working BW of 3.1–10.6 GH based on Federal Communications Commission (FCC) [38]. After adjusting the band of 0–10 GHz in a simulation process, the band of 1.2–7.2 GHz was obtained. It has been tried throughout the designing procedure to shift the antenna to the lower frequency band since the antenna is designed for the underwater communication applications.

During the designing procedure, some parameters exist, which play an essential role in antenna performances. The critical parameters list, as the patch and FL dimensions, the length of curved staircase GND (L_2–6_,L_11_), the length of the loading (Lg) at the back (these parameters construct and affect the UWB antenna performance initially), loading with stub (L_7–10_), shorting pins, and rectangular and arc slots etched form the patch (to improve the antenna radiation performances and increase the BW). An ordinary patch antenna with an elliptical shape is simulated at the center frequency of 5 GHz initially (the TL technique is applied to excite the antenna through the feed line (FL), and SMA port, and the formulas presented in Reference [39]). Then the antenna performances in terms of BW, gain, directivity, and radiation efficiency are investigated (in each step to design the patch and GND dimensions, which are optimized). All the equations and how to calculate the patch dimensions presented in References [40,41]. In addition to that, the patch dimensions alter both lower-end and higher-end of the UWB antenna’s BW as its major axis (a) has an impact on the lower-end of the BW, and the minor axis (b) offers a broader BW [39]. The working BW of the conventional UWB antenna started from 1.3 GHz and obtained the first pole at 1.7 GHz. The radiation efficiency of the antenna has a peak at 2 GHz by almost 100%. It reduced gradually between 2 GHz and 3 GHz. Afterward, the reduction continued steadily to 85% by the end of the frequency band. Furthermore, the antenna is radiated bidirectionally at most of the BW.

Afterward, the antenna’s ground is cut by a rectangular slot to make it staircase-ground and co-planar waveguide (CPW) feeding instead of using the usual transmission line method (L_2–6_, L_11_). This change on the ground increased the working BW and moved it to the lower frequency range slightly (having a staircase ground has already given an enhanced BW. Therefore, converting the edge of this staircase ground to a round shape increase the working BW along with the gain [42,43,44]). However, the radiation efficiency is decreased to 80% between 2.9 and 8 GHz and then increased to 90% at 10 GHz. The antenna is loaded from the back by a rectangular shape load with the length of Lg to make the radiation pattern of the antenna closer to an omnidirectional rather than a bidirectional with a narrow beamwidth. Although this loading improved the radiation pattern, some stop bands occurred in working bands. Hence, the antenna is loaded with three shorting pins. The locations of these pins chosen follow the surface current distribution (SCD). A loading antenna with these pins benefits us by removing the stop bands at 5.5–6.5 GHz along with reducing the working BW. The next step is to move the working BW more to the lower band and to improve the reflection coefficient level to have better impedance matching.

Therefore, the antenna is loaded again with a long stub with the length of L_7–10_. The loading stub with the length of (L_7-10_) is used to have the antenna resonating at the ISM band [45,46]. It shifts and reduces the frequencies. The actual length of the loaded stub is calculated according to the desired resonance (1.3 GHz). Furthermore, the location of this stub is chosen based on the SCD around the stub, patch, and the entire antenna. Apart from reducing the working BW by using the pins and the long stubs, the impedance bandwidth level is close to −10 dB at 2–3 GHz, and 4 GHz. Therefore, the rectangular and arc slots cut from the patch.

The space (*t_1_*) between the stubs L_7_ and L_8_ is optimized as to be neither too low nor too high to void degrading the radiation efficiency to avoid the destructive mutual coupling and surface wave (Figure 1). However, adding the stub to the junction gives a new resonance, but it enhances the surface waves around the intersection as well. Hence, the conventional rectangular GND is changed to a staircase shape to both avoid the degraded radiation efficiency caused by the coupling and the surface wave, and increase the BW. For further reduction of the surface wave and the undesired coupling, the arc-shaped slot with radius of *r*, and thickness of *t_2_* are cut from the patch. Cutting these slots changes the antenna polarization into a circular motion, which is important in mobile communications.

**Figure 2:**
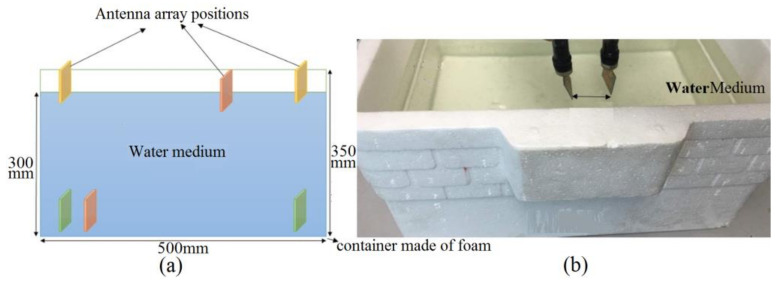
The system configuration in both simulation (**a**) and (**b**) measurement setups.

In addition to that, the slots are cut from the patch as capacitive loading to compensate the stopbands accrued after adding the stub and repress the stopband at around 4.8 GHz. Moreover, it can amend both the impedance bandwidth and the reflection coefficient level between 2 and 3 GHz. Figure 2 displays the antennas setup and arrangement for both a simulation and measurement in both mediums of air and water. Figure 1 depicts an elliptical patch antenna positioned on the X-Y plane with dimensions of 45 × 38 × 1.5 mm^3^.

The antenna is designed on a layer of a polytetrafluoroethylene (PTFE) substrate, which has $\varepsilon_{r}\;$ = 2.55, h = 0.8 mm (the thickness), and tan δ = 0.001 [47]. The proposed design of the elliptical patch was based on the optimized parameters for the best outcomes. The detailed parameters have the following variables: the dimensions of elliptical patch (a, b), dimensions of feed line (L_f_, W_f_), the stub’s lengths (L_7–10_), shorting pins location, and the curved staircase dimensions (L_2–6_, L_11_) (Table 1 shows the antenna dimensions). It was investigated in the medium of air initially. Consequently, the investigation was extended to its radiation characteristics in both mediums of fresh water and sea water (both measurement and simulation). An extra investigation was also performed on the mediums of distilled water and a Debye model water (simulation only) in a foam tank with dimensions of 50 cm × 35 cm × 30 cm. Besides, the S-parameters results present in terms of depth and distance between the antenna for both freshwater and seawater with a dielectric constant of 78 and 74, respectively. Basically, four types of medium utilities were simulated where two of them were also measured to demonstrate its capability while functioning, i.e., sending and receiving electromagnetic waves and signals.

When the optimization process is started, parameters which alter the lower and higher-end of the BW is assumed first as the semi-major axis, and the semi-minor axis of the patch disturbs the working BW and widening the BW, respectively. Then the feedline dimensions are optimized as its width has an impact on matching the antenna to the feed line.

The stubs, and the space between them and the patch, depends on the effects of the reflection coefficient result. It is beneficial because the space between the second part and patch becomes critical in terms of the negative coupling impacts. The slot width of t_1_ and t_2_ do not affect the impedance bandwidth outcome too much. Thus, these parameters were not depicted here.

**Figure 3:**
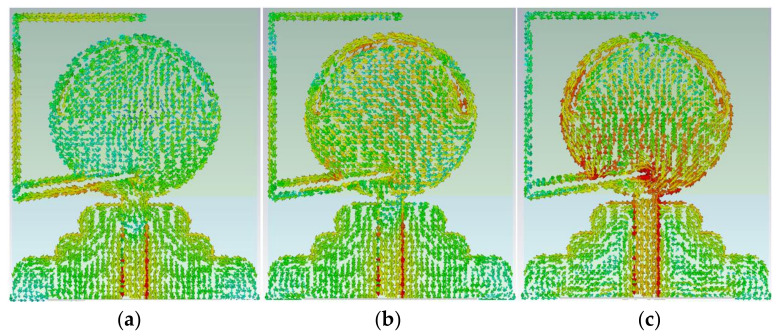
SCD result of the antenna at (**a**) 1.3 GHz, (**b**) 5 GHz, and (**c**) 7.2 GHz.

**Figure 4:**
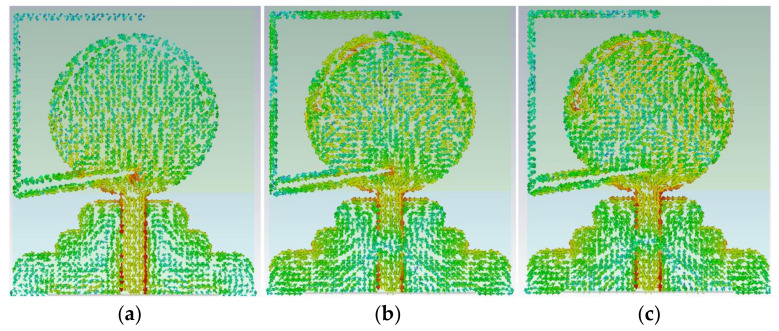
The antenna’s surface current distribution (SCD) result at poles in bandwidth (BW) at (**a**) 2.5 GHz, (**b**) 4.45 GHz, and (**c**) 6.8 GHz.

Four poles are attained in the reflection coefficient result at 1.7 GHz, 3.1 GHz, 4.4 GHz, and 6 GHz. Furthermore, to show how the wave is guided through the proposed antenna and analysis, Surface Current Density (SCD) of the patch, the stub is represented at frequency bands of 1.3 GHz, 2.5 GHz, 4.8 GHz, 5 GHz, 6.8 GHz, and 7.2 GHz, as shown in Figure 3 and Figure 4. UWB antennas act differently when compared to the narrowband antennas as they are supporting higher-order modes at higher frequency bands and fundamental mode at lower frequency bands. The major parts of the antenna, which are involved in a resonating call as the feed line, the patch resonator, the gap between the FL, and the staircase GND and the curved staircase GND itself. However, then, the current flow is changed and disturbed when it goes through the FL, patch, and the GND and its antenna performance (Figure 4).

As can be seen in Figure 3, the SCD shows more strength and intensity at the edge of the stub at 1.3 GHz, especially near the junction. Hence, Figure 3 confirms that most of the SCD density distributed around the stub at 1.3 GHz. The SCD tendency is the same as it has more density around the slots cut from the patch at the center frequency of 5 GHz (Figure 3). In addition to that, the SCD for higher-end of BW is depicted in Figure 3 at 7.2 GHz.

The SCD around the elliptical patch shows its critical role in making an ultra-wide BW. Figure 4 illustrates the SCD at other poles in the reflection coefficient result at 2.5 GHz, 4.45 GHz, and 6.8 GHz. The SCD density is high around the edge, the patch, and the feed line, and the arc shape slot. Moreover, this current density is not high at locations such as the stub, and the GND. As mentioned before, both the feed line and the patch affect the BW at its lower and higher end and enhance the BW. The slots that affected the stopbands occurred due to adding the stub (Figure 3 and Figure 4) [48].

It would be informative to show how the antenna functions in both time and frequency domains before starting the result and discussion section. The narrowband antennas and their propagation usually describe in the frequency domain, and their parameters are unchanged over a few BW. However, the frequency-dependent characteristics and their behavior should be investigated for UWB systems. In other words, since the UWB devices and systems are considered as a technology with an impulse-based nature, their time domain features should be known as well. Unlike the time domain in which the transmitting antenna resonates an impulse and the transient response of the antenna is adequate to describe the impulse system, the antenna transmits a continuous wave in the frequency domain [39].

## 3. Results and Discussion

### 3.1. Antenna’s Radiation Characteristics

After the simulation results of the antenna were obtained, the antenna is measured in the mediums of air and two types of water: freshwater and seawater. A Performance Network Analyzer (PNA) (model: E8363C, Keysight Technologies, Santa Rosa, CA, USA) is used to perform the measurement. The process starts with the calibration of the PNA, which is critical for attaining accurate results. Then, a frequency range of the PNA chooses a frequency between 500 MHz and 10 GHz, and 1002 samples. Then, during the measurement, the magnitude type is determined to observe the S-parameters (The same calibration type used by Reference [39] is used here). Two antennas used to measure the S-parameters in various mediums like fresh water and sea water are applied. Antenna 1 (Tx) is connected to the first terminal and the other one is connected to the second port PNA as the receiver (Rx). During the measurement, the Tx remains fixed, and the other antenna is moved to different locations in terms of space between the transmitter and receiver both horizontally and vertically (in depth). Finally, the S-parameters results are recorded for further analysis.

**Figure 5:**
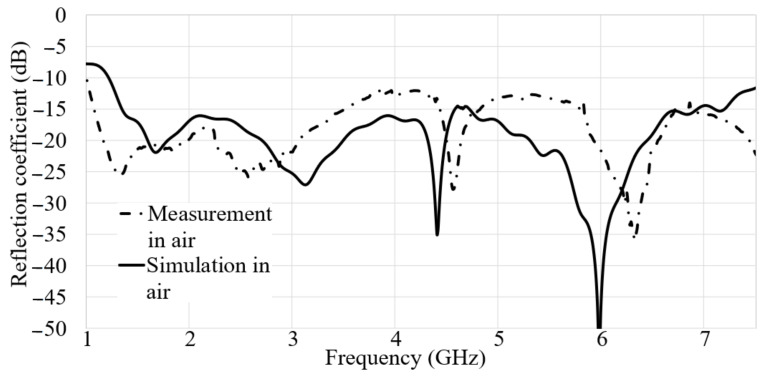
The S_11_ outcomes of simulated and measured assessment in air.

Figure 5 shows both the simulated and measured reflection coefficient results. According to the results presented in Figure 5, the measured and simulated reflection coefficient results are in good agreement. Besides, the obtained BW is acceptable as a result of a UWB antenna. The simulated and measured outcomes depicted in Figure 5 include a working BW of almost 6 GHz BW and a 138.8% fractional BW (FBW) is obtained. Therefore, it can be considered as a UWB antenna at the center frequency of 5 GHz.

Figure 5 clearly shows that the frequency bands are achieved at most of the working BW for the measurement result except a minor alteration from the simulated result. However, it still works. Furthermore, two stopbands occurred in the measurement result as 3.8–4.45 GHz and 5–5.6 GHz. The differences and tolerances that occur after fabrication of the antenna cause the differences between the simulated and measured reflection coefficient result. The simulation and measurement conditions are not similar. For instances, in CST, a waveguide port is used through the macros section to feed the antenna using the substrate thickness and the FL width. On the other hand, the SMA port, which is utilized to connect the antenna to the coaxial cable has a center pin that can cause a significant radiation. Thus, the pin should be short enough to reduce this unwanted radiation, especially at higher frequencies. Besides, they showed that the shorter pin of the SMA connector gives a smaller Voltage Standing Wave Ratio (VSWR) [49]. In addition to that, for the length of 1 mm, the soldering will be more comfortable, and it should not raise the resistivity in the GND layer.

**Figure 6:**
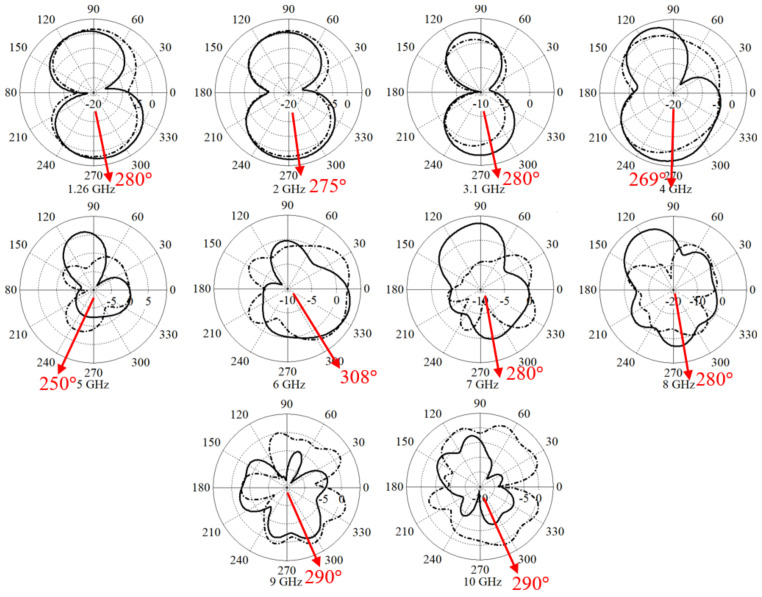
E-field and H-field of the far-field radiation pattern.

Figure 6 presents the E-field and H-field components of the far-field radiation pattern at θ = 0 and 90°. Since the reflection coefficient result of simulation and measurement are in good agreement, the simulated radiation pattern can be trusted. The far-field gain at the frequency range of 1.26–10 GHz is depicted in Figure 6. Their values are 0.35 dBi, 0.6 dBi, 0.9 dBi, 1 dBi, 2.2 dBi, 3.56 dBi, 4 dBi, 4.4 dBi, 4.85 dBi, and 6.02 dBi [50].

The main district of the lobe is observed between 280° and 300° at 1.3 GHz in Figure 6. The radiation pattern tendency remains unchanged when it reaches the frequency range of 5–6 GHz. It is altered by approximately 30 degrees to 250° at 5 GHz. Then, it continues to 300° to 6 GHz. Afterward, the pattern is rotated toward 280° until the end of the band at 10 GHz. The main district lobe showed a red arrow in Figure 6. As previously mentioned, since the simulated and measured reflection coefficient shows good agreement, the simulated radiation pattern can be reliable. Furthermore, the antenna has a maximum gain and directivity of 6.02 dB and 7.09 dBi, respectively. The radiation efficiency of the antenna is an important parameter of the antenna and it is 55%, 90%, 85%, and 90% at 1.3 GHz, 2 GHz, 7 GHz, and 10 GHz, respectively.

**Figure 7:**
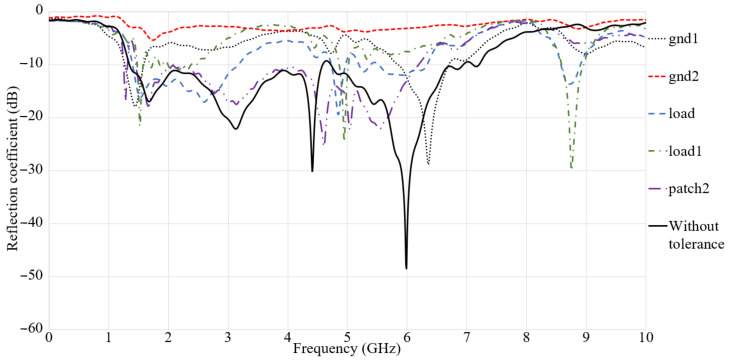
S_11_ result of the antenna in terms of fabrication tolerances.

The antenna has a minute structure, which is comprised of stubs and slots. Hence, it is advised to examine the accuracy of fabrication to avoid antenna’s sensitivity in terms of possible altered results due to fabrication defects (Figure 7).

As can be seen in Figure 7, the S_11_ result shows the results in comparison to the time that any tolerances or misalignment occurs vertically or horizontally. Furthermore, Figure 7 shows that any misalignment in the antenna will change impedance matching of the antenna less or more (GND 1: 2 mm, GND 2: 1 mm, load: 2 mm, load 1: 1 mm, patch 2: 2 mm). Some stopbands occur in the working BW due to these misalignments’ tolerances after fabrication. The GND 1 (ground misalignment to the left), GND2 (ground misalignment to the right), load (the loading at back misalignment to the left), load 1 (the loading at back misalignment to the right), and the patch show that the misalignment occurs after fabrication.

### 3.2. Antenna Characteristics in Air and Water

Figure 2 demonstrates the measurement and simulation setup in air and water. Furthermore, the water foam tank’s dimensions are 50 cm × 35 cm, and the water depth is 30 cm. The same procedure is done in simulation, following in the measurement of the antenna investigations. According to the antenna locations shown in Figure 2, one antenna is kept fixed and the second one is flexible to move horizontally and vertically. Then, the antenna characteristics are measured for a different space and depth.

**Figure 8:**
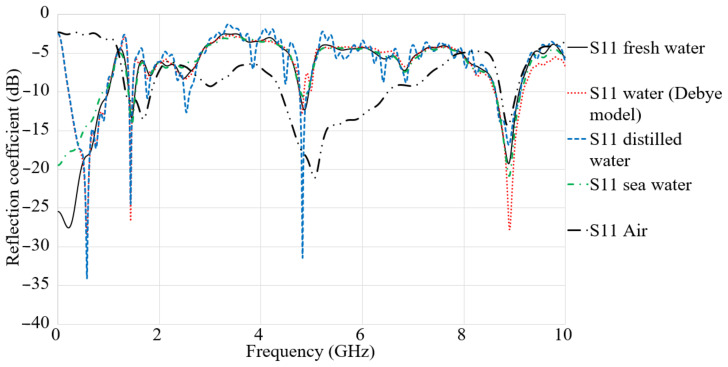
Simulated S_11_ results in air, fresh water, water (Debye model), sea water, and distilled water.

Figure 8 expresses the simulated S_11_ results in fresh water, water with the Debye model, distilled water, and seawater. The variation in the result shown in Figure 8 are neglectable, and the ultra-wide BW is changed to a multi-band. As can be seen in Figure 8, the reflection coefficient results depict the best results for the distilled water, which obtained more working resonances in the BW. The other kinds of water used in this evaluation follow a similar trend. The reflection coefficient results depict a better level for distilled water, which is followed by water with the Debye model. Furthermore, seawater illustrated the lowest BW in comparison with others. The alteration on the S-parameter results of the antenna in different distances is because of the time that takes the signal to reach the receiver. The time delay increases with the gap between the transmitter and the receiver. In addition to that, it can be concluded that this time delay is dependent on the size of the foam container. Thus, a taller media degrades the reflection coefficient result. Based on the results shown in Figure 8, the air has the best result of a reflection coefficient due to its lowest relative permittivity in comparison with the other kinds of water. Unlike the reflection coefficient result of the air, the results for other types of water did not differ much because of their similar relative permittivity. Among all the kinds of water, the distilled water illustrates better outcomes.

**Figure 9:**
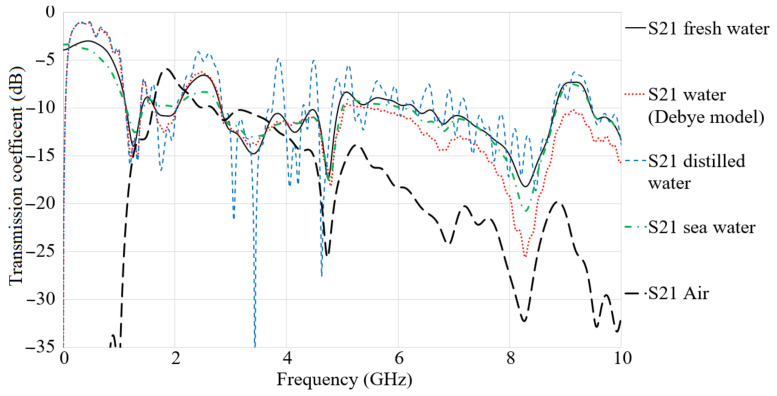
Simulated S_21_ results in air, fresh water, water (Debye model), sea water, and distilled water.

Figure 9 depicts the simulated S_21_ results in air, fresh water, water (Debye model), and distilled water.

The transmission coefficient follows similar trends for all types of water and air. It shows the higher level at a lower band until 4 GHz and then decreased gradually until the end of the band in air medium. The transmission response of freshwater, the Debye model, and seawater presents satisfactory results until 5 GHz, while the freshwater has the highest level at most of the frequency band, which includes the level followed by sea water and the Debye model water. After 5 GHz, the results are degraded by −10 dB, −20 dB, and −25 at 8 GHz for fresh water, sea water, and water with the Debye model, respectively. The results in the air are better than other mediums at most of the working BW. The mediums other than air like different kinds of water have higher permittivity. Hence, the signal cannot pass through these mediums as quickly as the air medium and degradations. In order to understand the dielectric constant and the relative permittivity, it is better to clarify that the dielectric constant plays a critical role when it comes to impedance consideration, which correlates with the frequency, and it shows a reduction with frequency enhancement, but this dependency varies in different materials. For example, those materials that can be constant for a broad frequency range can be considered as suitable for high-frequency applications. Another vital factor is a dielectric loss tangent or a dissipation factor. The tangent loss of material shows how much power is lost as the loss tangent of the antenna substrate is tried to as low as possible when choosing the antenna’s substrate. The tangent loss of material shows the power lost because of the material [39].

**Figure 10:**
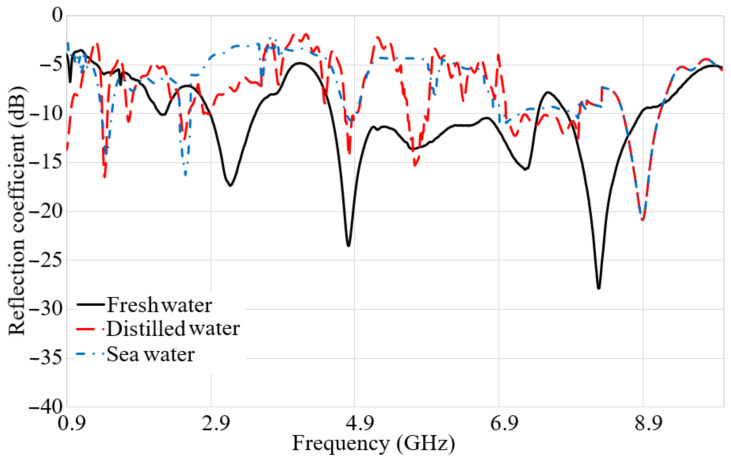
Measured S_11_ result in fresh water, distilled water, and sea water.

**Figure 11:**
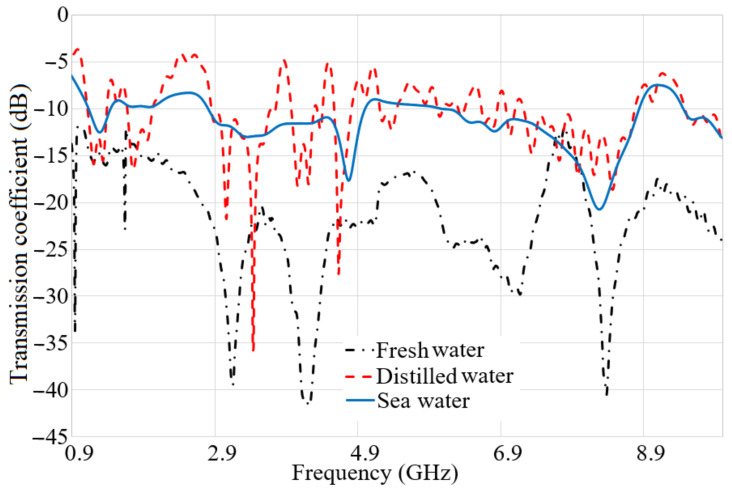
Measured S_21_ result in fresh water, distilled water, and sea water.

The S_21_ result shows that it is better not to have ripples and fluctuations in order to show a low distortion of the signals when it penetrates the water [51,52]. Figure 10 depicts the S_11_ result in fresh water, distilled water, and seawater. Better impedance matching is obtained for fresh water, as presented in Figure 10. The working frequency band for distilled water are more than seawater, and the reflection coeffect level is lower than seawater, although both seawater and distilled water are following a similar trend for most of the band. In addition to that, the fresh water shows a broad BW between 3.5 GHz and 7 GHz. The measurement and simulation setup in Figure 2 are followed to measure the transmission coefficient of the antenna (Figure 11). Two antennas are located at two different sides of the water tank with dimensions of 50 cm × 35 cm and a water depth of 30 cm with the distance up to 50 cm and a depth up to 30 cm to measure S-parameters, as presented in Figure 2. The S_21_ difference is reduced by more than 10 dB for a measurement in fresh water in comparison with distilled water and sea water. The transmission coefficient result for distilled water shows a ripple, unlike the results for sea water. Furthermore, the transmission coefficient result for distilled water illustrates a higher level compared to the sea water and its variations are less than 10 dB, especially 5 GHz to 8 GHz. The sea water did not show ripples in the result, and it has the same tendency as distilled water if the ripples are ignored. It is understandable that the transmission coefficient result is dissipated after 10 GHz and onward since the degradation has already started at 9 GHz. The S_21_ decrement and the occurred distortion are because of the shorter wavelength at a higher frequency, and the higher $\varepsilon_{r}$ of fresh water and distilled water in comparison with air [53].

### 3.3. Antenna Characteristics in Different Distances and Positions

After investigation of the antenna in different types of water, two antennas are used to investigate the proposed antenna when the antenna is in different distances and depth, as are both at the surface of the water, including one at the surface and the other submerges the water, and when both antennas are submerged in the water. In this step of the study, the distance between the two antennas were altered (the depth is constant and near the bottom of the tank) and then their effects on the reflection coefficient and transmission coefficient were examined.

**Figure 12:**
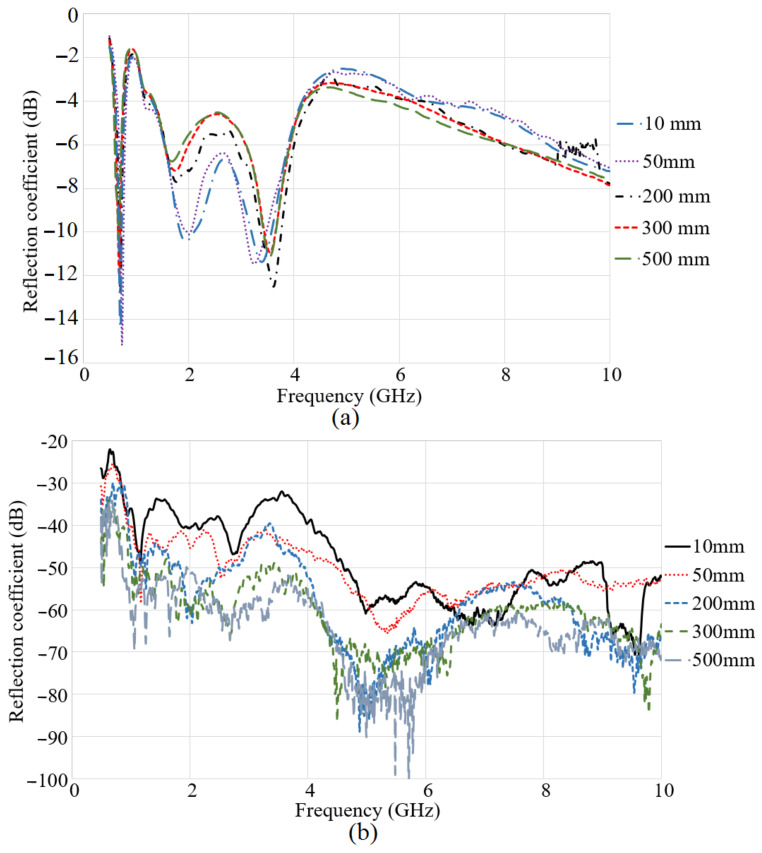
The reflection (**a**) and transmission coefficient (**b**) results when one antenna is at the surface and another is submerged.

Figure 12 shows the S_11_ and S_21_ results when the receiver is submerged in the water at a depth of 30 cm, and the other antenna (the transmitter) is kept at the surface of the water.

Since the dielectric properties of the water samples used in the previous part of the study did not differ too much, only fresh water was used as the medium at this stage of the research. It shows that the obtained wideband for the antenna in the air is considered to convert it to a triple band when it is in the water medium. When the space between the antennas is 10 mm, three resonances are obtained around 1.3 GHz, 2 GHz, and 3.6 GHz. These three resonances are still working at the distance of 180 mm. When the gap between the antennas exceeds 180 mm, the second resonance started to reduce its level, and this trend keeps on reducing to a distance of 500 mm. In addition to that, the variation of distance does not affect the first and the third resonances too much. However, the same tendency of degrading in the reflection coefficient level follows in the first and third frequency bands, but they are still working even up to 500 mm. The right side of Figure 12 shows the transmission coefficient result of the system at different distances. It is presented that, when the distance is 10 mm, the transmission coefficient level is at its highest level and it has a higher level to the center frequency of 5 GHz, and, after 5 GHz, it is reduced by 10 dB. By increasing the distance from 10 mm to 50 mm, the transmission coefficient level is degraded by almost 10 dB again. Furthermore, increasing the space between the transmitter and receiver in the system affects the transmission coefficient level in the same movement as when the distance was less than 200 mm, and the reduction continues to 500 mm.

**Figure 13:**
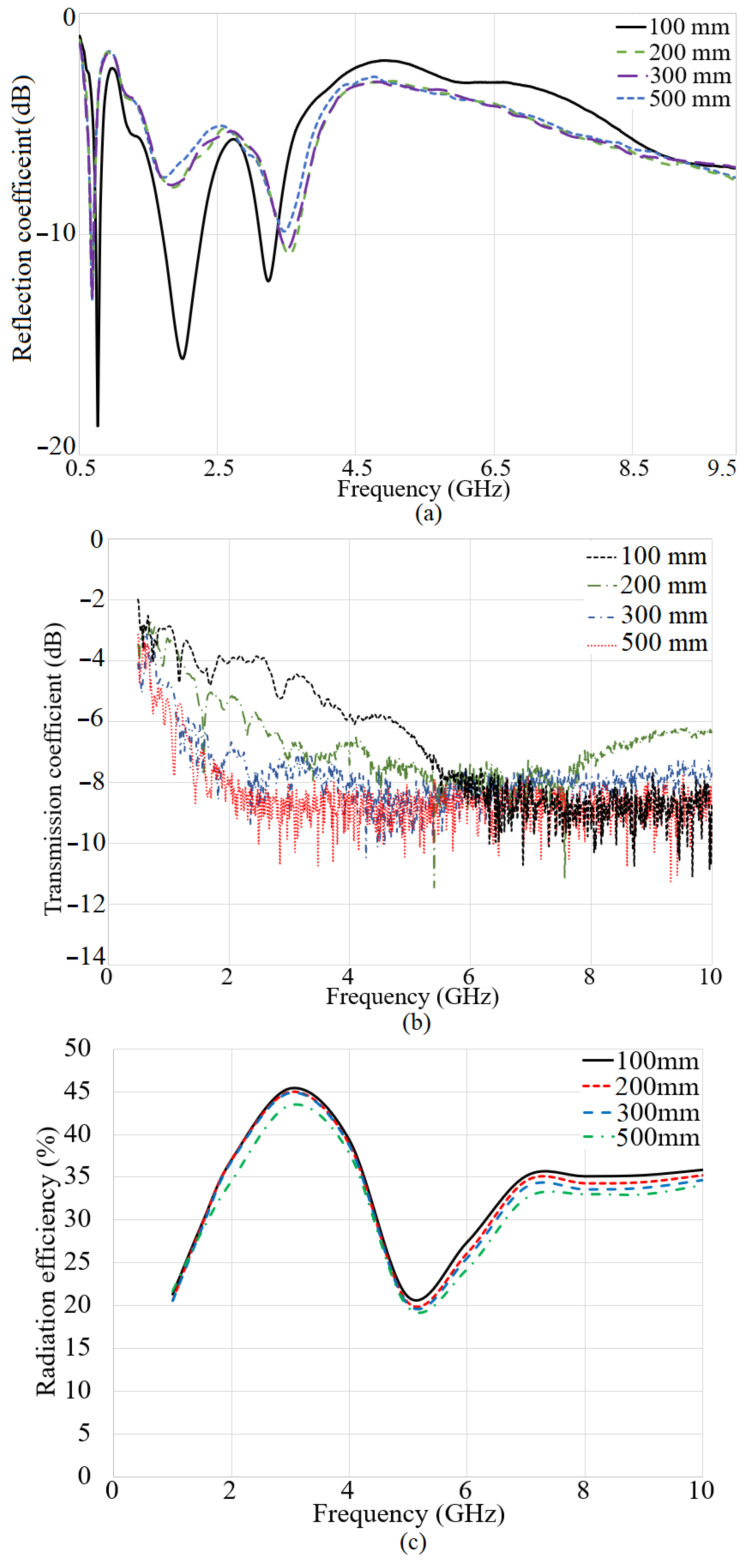
The reflection coefficient (**a**), transmission coefficient (**b**), and (**c**) radiation efficiency results when both antennas are submerged.

The S-parameter results of the system were presented in Figure 13, when both antennas are submerged. The left side of Figure 13a shows the S11 result in terms of the distance when the transmitter and receiver are in a depth of 30 cm. When the space between the transmitter and receiver is 100 mm and less, three working bands are attained. Furthermore, exceeding the space from 100 mm, reduces the level of the reflection coefficient at the second resonance. Besides, enhancing the space between the antennas causes a reduction in the second band further.

Moreover, Figure 13b illustrates the transmission coefficient result of the system when both the receiver and transmitter are submerged in the water medium in the space up to 500 mm. The transmission coefficient result depicts that the transmission coefficient has the highest level when the space between the antennas is 100 mm or less. Enhancement in the distance decreases the transmission coefficient level at most of the frequency band. At all the gaps, the transmission coefficient level is degrading with the frequency enhancement. In addition to that, the transmission coefficient level is higher as compared to the time that one antenna was at the surface and the other one was submerged. Figure 13c depicts the radiation efficiency results of the antennas when both antennas are immersed. It indicates that the radiation efficiency results follow the same tendency as transmission coefficient results, and it reduces with distance. Additionally, it has a peak at around 45% near the lower band of the working BW. Then, it reduces to 22% at 5 GHz and again jump to 35% at 6.5 GHz. Afterward, it becomes constant. Overall, the radiation efficiency of the antenna for all distances are more than 20%.

**Figure 14:**
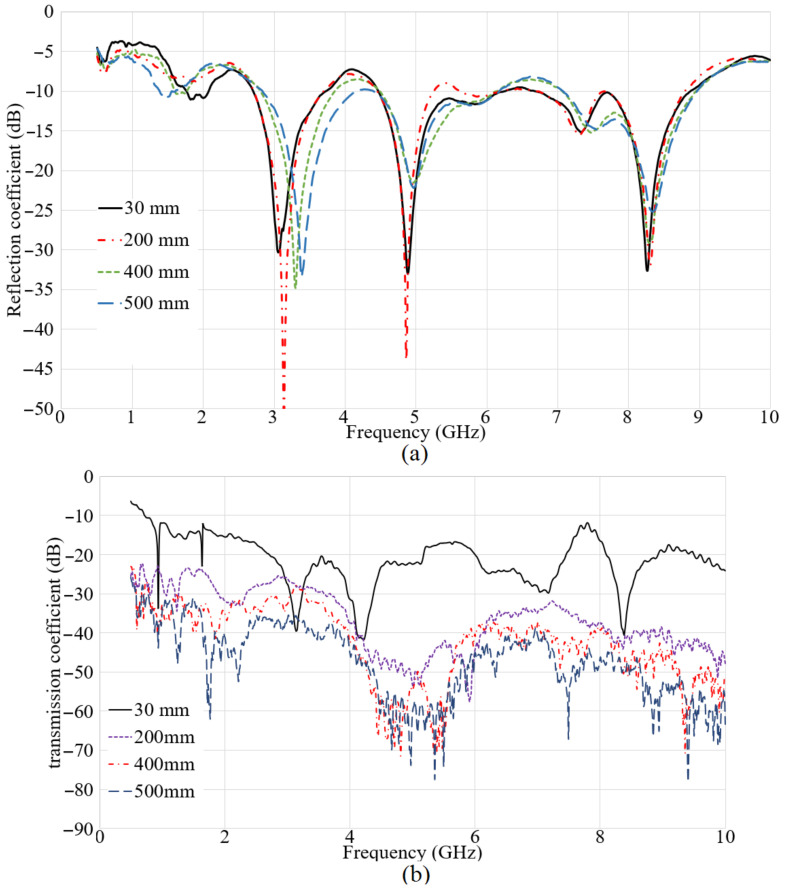
The reflection (**a**) and transmission coefficient (**b**) results when both antennas are at the water surface.

Figure 14 portrays the S_11_ and S_21_ results when both the transmitter and receiver were at the surface of the water. When both antennas were at the surface of the water, the lower part of the working BW obtained in the air is converted to a stop-band except from 1.4 GHz to 2.2 GHz. Besides, other band-stops occurred at 4.2 GHz and 6.8 GHz at 30 mm and less. When the space was enhanced, the working bands and poles are shifting to the higher band. This enhancement changes the stop-bands and shifts them. Additionally, the S_21_ result shows the highest level for the lowest distance at 30 mm. Furthermore, its level is followed by the other spaces at 200 mm, and it is degraded by distance.

### 3.4. Communication Performance of the Antenna System

To show whether the proposed antenna operates well in a water environment, the antenna performance in terms of channel capacity, bit error rate (BER), and data rate are calculated, measured, and compared for three locations (both antennas at surface, both antennas submerged, one at the surface, and one submerged) and two types of water (the communication performance of the antenna system was investigated using MATLAB software after the data was extracted from Computer Simulation Technology (CST) microwave studio simulator). The data rate transmission calculation is essential to both types of Single Input Single Output (SISO) and Multiple Input Multiple Output (MIMO) communication systems. This calculation is one of the fundamental factors of investigating a network capability to send a signal from the receiver to transmitter. All researchers working in information theory know about Shannon’s theory and how to calculate the channel capacity [54,55,56].

**Figure 15:**
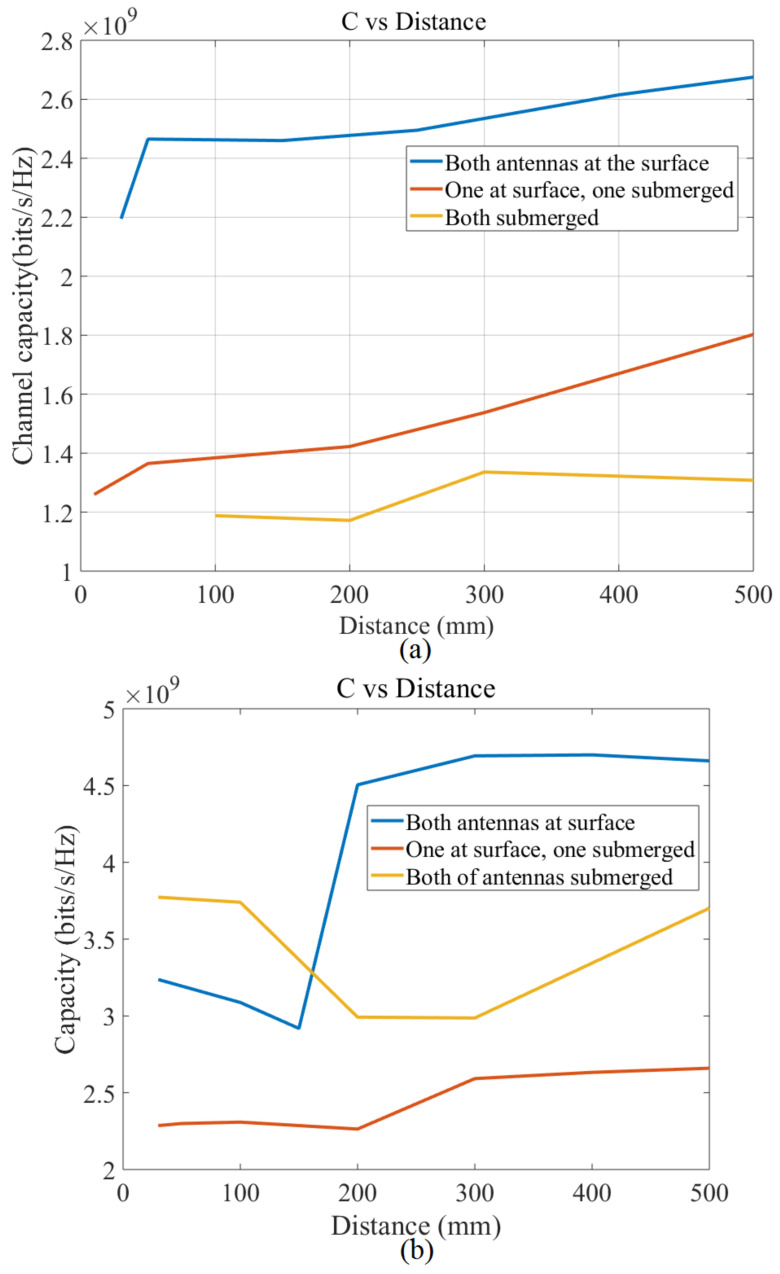
Channel capacity in terms of the distance between the antennas with constant bandwidth (BW) (**a**) fresh water, and (**b**) sea water.

Figure 15 shows the channel capacity in terms of the distance between the antennas for three different positions for both freshwater (left) and seawater (right) (the BW is assumed constant). The results follow a similar trend in both types of water.

Furthermore, in both types of water, the channel capacity has the highest level when both antennas are located at the surface of water. But, for the other two antenna positions (one at the surface and one submerged, both submerged), the trend changed in different types of water. For instances, when one antenna is located at the surface of fresh water and the other one is submerged, the channel capacity has a higher level than when they are both submerged in freshwater. On the other hand, the channel capacity level of the positions’ orders is different than when they are submerged in seawater. When the type of water changed to seawater, the channel capacity has a higher level for both antennas submerged than when one of them is at the surface, and one is in a deep level of water/submerged.

**Figure 16:**
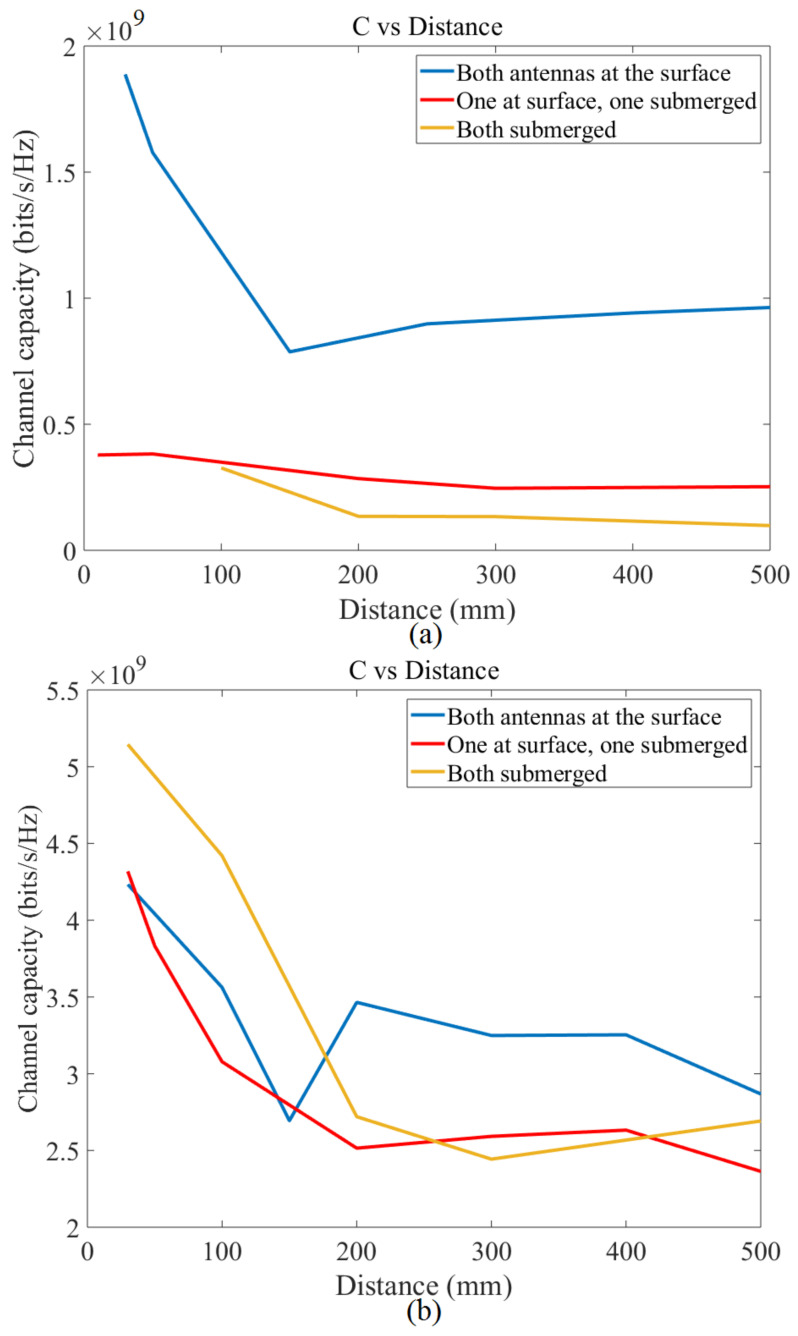
Channel capacity in terms of the distance between the antennas with a maximum bandwidth (BW) (**a**): fresh water and (**b**): sea water.

Figure 16 presented the channel capacity in terms of the distance between the antennas with maximum BW of the proposed UWB antenna in two different kinds of water. Furthermore, it shows the reduction of the channel capacity with an increased distance when the maximum BW is essential. In addition to that, it shows that, when the space between the transmitter and receiver enhances, the channel capacity is decreased. It happens due to the decrease in maximum BW of the proposed antenna when the distance raises (the BW alteration is clearly shown in Figure 10). Besides, the channel capacity has a higher level when both antennas are at the surface, and it follows by two other situations such as one surface/one submerge, and both submerge.

**Figure 17:**
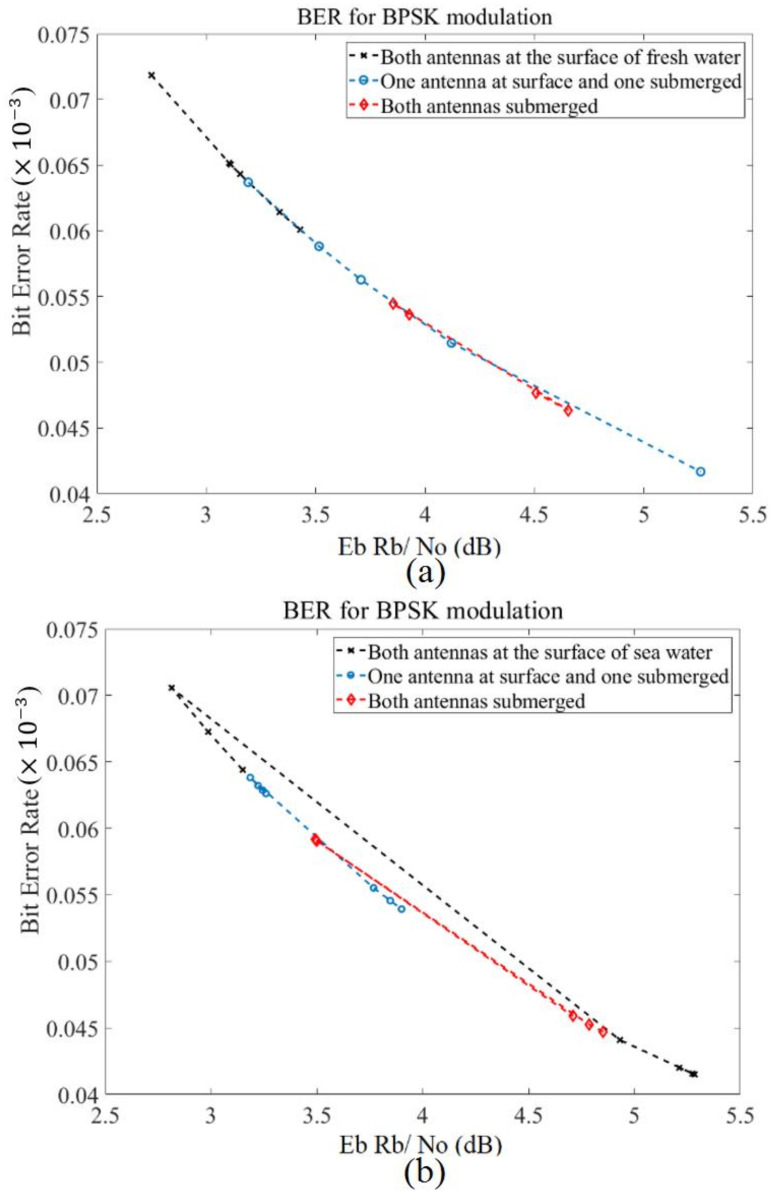
A bit-rate R_b_ average energy per bit E_b_ and noise power density N_0_ in terms of BER (**a**) fresh water, and (**b**) sea water.

Figure 17 illustrates the Bit Error Rate (BER) in terms of bit rate average energy $E_{b}R_{b}/N_{0}$ in both freshwater and seawater at three different locations. In order to show the proposed antenna is working well for underwater communications, the BER using a Binary Phase Shift Keying (BPSK) modulation is shown in terms of the average energy in two kinds of water for three various locations. A similar tendency is found for both types of water. The BER in freshwater shows a reduction with an increase in average energy, and it has the highest level when both antennas are at the surface. Changing the media to seawater alters the tendency slightly. However, the trend is the same for most of the power intensity.

**Figure 18:**
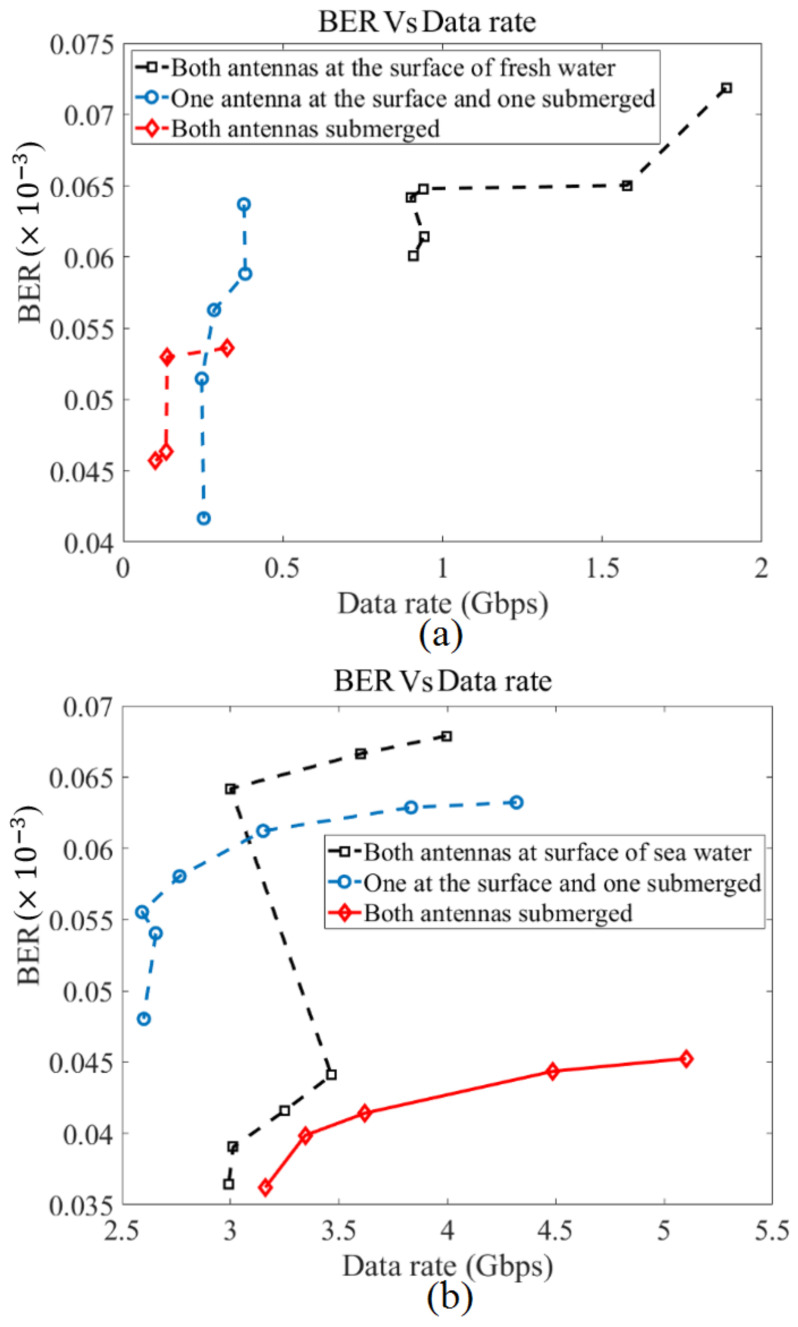
The BER variations in terms of data rate (Gbps) (**a**) fresh water, and (**b**) sea water.

Figure 18 illustrates the BER in terms of the data rate in three different locations for two types of water (fresh water and sea water). In both types of water, the tendency goes as BER increases with a data rate increment. Furthermore, the BER level is at its highest level when both antennas are at the surface of the water, and the lowest level when both are submerged. In addition to that, at a data rate of 1.8 Gbps, a BER of 0.0725 × 10^−3^ is measured in fresh water, which passes the Forward Error Correction (FEC) limit. Furthermore, the BER level is degraded when the media changes to seawater as, at a data rate of 4 Gbps, the BER is almost 0.067 × 10^−3^. These are consistent with the presented simulation calculations (only the measured data presented here). Furthermore, to improve transmission, the equalizer can be useful [57].

## 4. Conclusions

A compact elliptical patch microstrip 5G UWB antenna is presented for underwater communication applications. The proposed antenna uses an elliptical patch, a feed line, and loading the antenna with a stub connected to the junction of patch and feedline (to move the whole working BW to the lower band and it has an Industrial, Scientific, and Medical (ISM) band working), plus slots to make the antenna have a circular polarization to improve the communication in different mediums of water (the antenna loadings removed the stopbands to make the communication at sub 6 GHz bands possible). Antenna shows a wide working BW between a 1.3 GHz and 7.2 GHz frequency band and fractional BW of 138.8% (f_1_ = 1.3 GHz, f_2_ = 7.2 GHz, f_3_ = 4.25 GHz). Furthermore, the 3 dB beamwidth at almost 280° at most of the BW is almost stable but at a slight alteration. In addition to that, a maximum gain of 6.02 dBi and directivity of 7.09 dBi are achieved. The simulated ad experimental S_21_ results depicted low ripples and fluctuations (almost 5 dB around 1.3–7.2 GHz) when both antennas were submerged. Therefore, low-level distortions in transmitted and received signals are expected.

The proposed UWB antenna can be a reliable candidate for underwater communications since a good agreement exists between the simulated and measured reflection coefficient in various environments. Accordingly, the antenna was simulated and measured in two types of water and three different positions (both at the surface of the water, both submerge with one at the surface and one immersed) to prove that it really functions underwater.

For three different locations of antennas in water, the performance is critically evaluated in terms of channel capacity, error-rates, and the data-rates. For constant BW, the antennas placed at the water surface provide much higher capacity compared to those immersed in the water. The presence of salt, temperature, air bubbles, and colored dissolved organic materials (CDOMs) in sea water increases the capacity up to 4.7 Gb/s/Hz. Increasing the distance between antennas also widens the capacity with constant BW utilization, but vice versa is true for maximum BW usage. An increase in the average energy reduces the BER. However, the antenna locations do not make a difference in terms of BER versus SNR. The usage of transceivers at the surface of fresh water can yield data rates up to 1.8 Gbps. However, no significant data rates can be achieved when one or both antennas are immersed in water. Yet, the transceivers immersed in seawater can provide the transmission with data rates of 5.2 Gbps.

The compact elliptical UWB-based transceiver’s performance was evaluated in terms of the capacity, the error-rates, and the data-rates, with three possibilities given (i.e., both antennas are at the surface, any of the antenna is at the surface, both antennas are immersed into water) for both types of fresh and sea water. For different antenna locations and water types, an increase or decrease in performance was observed. The surface mounted antennas provide high data rates in both types of water, while the average energy minimized the BERs. The transceivers immersed in seawater can provide data rates up to 5.2 Gbps using compact UWB antennas. Based on the achieved results from both simulated and measured investigations, the antenna can be an adequate candidate for underwater communication applications as ocean biology, environmental research, surveillance, seismic monitoring, ship hull monitoring, communicating with submarines, diver communications, monitoring pollution in a water environment, collection of data from the bottom of the sea, detection of new objects, and transmission of data between the ships.

## Figures and Tables

**Table 1:** The optimized dimensions of the proposed antenna.

Parameters	Values (mm)	Parameters	Values (mm)
L_s_	45.00	L_2_	5.90
W_s_	38.00	L_3_	3.40
a	12.00	L_4_	6.50
b	13.00	L_5_	4.62
L_g_	29.00	L_6_	3.48
t_1_	1.43	L_7_	12.30
t_2_	1.00	L_8_	8.60
L_f_	16.00	L_9_	27.50
W_f_	2.35	L_10_	20.00
L_1_	17.00	L_11_	7.10

## Data Availability

Not applicable.
